# Confounding and missing data in cost-effectiveness analysis: comparing different methods

**DOI:** 10.1186/2191-1991-3-8

**Published:** 2013-03-28

**Authors:** Tommi Härkänen, Timo Maljanen, Olavi Lindfors, Esa Virtala, Paul Knekt

**Affiliations:** 1 National Institute of Health and Welfare, Mannerheimintie 166, P.O.Box 30, FIN-00271 Helsinki, Finland; 2 Social Insurance Institution, Helsinki, Finland

**Keywords:** Clinical trial, Cost-effectiveness analysis, Confounders, Predictive margins, Two-part model, Bayesian inference

## Abstract

**Introduction:**

Common approaches in cost-effectiveness analyses do not adjust for confounders. In nonrandomized studies this can result in biased results. Parametric models such as regression models are commonly applied to adjust for confounding, but there are several issues which need to be accounted for. The distribution of costs is often skewed and there can be a considerable proportion of observations of zero costs, which cannot be well handled using simple linear models. Associations between costs and effectiveness cannot usually be explained using observed background information alone, which also requires special attention in parametric modeling. Furthermore, in longitudinal panel data, missing observations are a growing problem also with nonparametric methods when cumulative outcome measures are used.

**Methods:**

We compare two methods, which can handle the aforementioned issues, in addition to the standard unadjusted bootstrap techniques for assessing cost-effectiveness in the Helsinki Psychotherapy Study based on five repeated measurements of the Global Severity Index (SCL-90-GSI) and direct costs during one year of follow-up in two groups defined by the Defence Style Questionnaire (DSQ) at baseline. The first method models cumulative costs and effectiveness using generalized linear models, multiple imputation and bootstrap techniques. The second method deals with repeated measurement data directly using a hierarchical two-part logistic and gamma regression model for costs, a hierarchical linear model for effectiveness, and Bayesian inference.

**Results:**

The adjustment for confounders mitigated the differences of the DSQ groups. Our method, based on Bayesian inference, revealed the unexplained association of costs and effectiveness. Furthermore, the method also demonstrated strong heteroscedasticity in positive costs.

**Conclusions:**

Confounders should be accounted for in cost-effectiveness analyses, if the comparison groups are not randomized.

**JEL classification:**

C1; C3; I1

## Background

Cost-effectiveness analyses are often based on comparing average costs with the effectiveness of the treatments, and on bootstrap methods [1]. Bootstrap methods have been shown to have good properties when compared with parametric methods[2-4]. A standard application of this approach does not adjust for possible confounding effects, which can have a considerable influence on the results not only in observational but also in randomised studies [5]. In randomized controlled trials spurious differences in the distributions of background factors can emerge due to random variation especially with small sample sizes. This can induce a need to adjust for confounding. Some statistical methods have been developed to estimate adjusted means using (generalized) linear models, but it seems that these methods have seldom been applied in cost-effectiveness analyses. In the predictive margins approach [6,7] the mean of individual predictions based on a generalized linear regression model are calculated, which allows for comparison of different scenarios by modification of the covariate values. In Bayesian inference, predictive distributions (e.g. [8]) can be applied for calculating adjusted means.

Longitudinal data have often been compressed by using different cumulative measures of costs and effectiveness outcomes (e.g. area under curve, AUC). The application of cumulative costs can also reduce the number of observations with zero costs in repeated measurement data and skewness, therefore, reducing the problems related to the application of linear models.

A drawback of cumulative measures is that a missing value in the outcome variable at a given measurement point will result in missing values in the cumulative outcome values of the subsequent measurement points. Multiple imputation (MI) [9] and data augmentation [10] can be applied to deal with missing outcome values at single measurement points, so that all of the information for the observed outcome values can be utilised for the cumulative outcomes.

The distribution of costs is generally skewed, with a proportion of observations having zero costs and the remaining portion having positive costs. Direct application of linear regression models is not suitable for these kinds of data, because if the model is used to predict costs, the predictions could suggest negative costs or other unrealistic results in some scenarios. The usual method of reducing skewness using logarithmic transformation is not sensible when some of the study subjects have zero costs. Two-part models [11], for example, have been proposed for solving this problem.

Missing values may be present not only in the dependent variable but also in the independent variables in the regression model. These variables can be, for example, skewed or categorical. Multiple imputation of missing costs with possibly zero values is more complicated, and two-part models can be more useful. In these cases, methods which assume a multinormal distribution for the variables are not ideal. More suitable methods, which can handle the aforementioned complications, can be based on data augmentation and Bayesian inference and implemented, for example, by using the OpenBUGS software [12,13].

In the case of an observational study and non-intervention-based comparison groups based on, for example, a patient characteristic the cost-effectiveness analysis demonstrates the change of the average costs (relative to the change in the average effectiveness). This information allows a decision-maker to plan more cost-effective interventions, as not only different patient-specific characteristics but also other baseline factors can be compared in terms of the standardized average cost differences.

The Helsinki Psychotherapy Study (HPS) is a randomised clinical trial comparing three therapy treatments [14]. In addition to comparisons of the randomized therapy groups, the data set can be utilized to conduct cost-effectiveness analyses by comparing groups defined by nonrandomized baseline factors, in which case the randomization no longer plays any part and confounding factors generally need to be adjusted for. In the present work, we compared two groups based on the Defence Style Questionnaire (DSQ) [15], resulting in the identification of several potential confounders.

The aims of the study were as follows: a) We handle confounding by applying predictive margins in the frequentist inference and predictive distributions in the Bayesian inference to produce adjusted means of cost and effectiveness, and their differences. b) We address missing data at single measurements of a repeated measurement study by using the multiple imputation and data augmentation techniques. c) We assess the unexplained associations between costs and effectiveness by applying Bayesian hierarchical models. d) We handle nonnegative costs by applying a two-part model using a logistic regression model as an indicator of zero or positive costs, and a hierarchical gamma regression model for positive costs to avoid unrealistic negative predictive costs, which can be a result of an application of simple linear models. Effectiveness is analysed by using a hierarchical linear model.

## Methods

### Data

The Helsinki Psychotherapy Study recruited 326 outpatients, aged 20–46 years, from the Helsinki region over the period 1994–2000 [14]. A statement describing explicitly the ethical background to this study and an approval by the Helsinki University Central Hospital’s ethics council can also be found in [14] (p. 31). Of these patients, 101 were randomly assigned to short-term psychodynamic psychotherapy, 97 to solution-focused therapy and 128 to long-term psychodynamic psychotherapy. In the present study, we restrict our analyses to the former two groups containing 198 patients having received short-term therapies. Of these patients, 7 refused to participate after being assigned to the treatment group and 21 discontinued their treatment. The cost-effectiveness analysis of the intervention groups has been reported elsewhere [16].

The measurement points (MP) at which the patients were measured were the baseline, and 3, 7, 9 and 12 months after the start of therapy. The Defence Style Questionnaire (DSQ) was dichotomized using the median value of 4.0 as the threshold. A total of 93 patients had DSQ <4, 100 had DSQ ≥4, and 5 had a missing DSQ value.

The effectiveness measure was the Global Severity Index, SCL-90-GSI, psychiatric symptoms, hereafter abbreviated as GSI [17]. The cost variable of the incremental cost-effectiveness analysis was the direct costs resulting from psychiatric health problems (DCP) during the 12-month follow-up period. The DCP was the sum of seven cost items in euros (€), which were: the costs accruing from 1) the protocol-driven study of psychotherapy treatments, 2) auxiliary study treatment visits, 3) other psychotherapy sessions, 4) outpatient visits to physicians and to other health care personnel concerning mental health problems, 5) inpatient care in psychiatric hospitals or with psychiatric diagnosis, 6) psychotropic medication, and 7) travel costs due to therapy visits. All costs were included in the analysis regardless of the payer. Effectiveness was assessed at baseline and at 3, 7, 9 and 12 months after baseline. Cost data based on information obtained from patients by questionnaires covered periods 0–7 months and 8–12 months whereas cost data based on patient level registers covered periods 0–3, 4–7, 8–9 and 10–12 months. Table 1 presents descriptive statistics of the outcome variables.

**Table 1:** Descriptive statistics of outcomes

		**DSQ, immature defence styles**								
		**DSQ <4 (N=93)**					**DSQ ≥4 (N=100)**			
**Variable**	**Month**	**Mean**^**a**^	**SD**^**b**^	**NZ**^**c**^	**NM**^**d**^		**Mean**^**a**^	**SD**^**b**^	**NZ**^**c**^	**NM**^**d**^
GSI	3	0.85	0.51		7		1.19	0.55		11
GSI	7	0.70	0.48		14		1.08	0.60		19
GSI	9	0.64	0.45		10		1.02	0.63		22
GSI	12	0.65	0.51		13		1.01	0.62		21
AUC of Eff	3–12	0.51	0.31		18		0.8	0.39		30
Costs	0–3	735	251	0	9		832	313	0	15
Costs	3–7	689	309	1	9		802	627	1	15
Costs	7–9	185	259	38	9		234	309	31	19
Costs	9–12	186	345	41	9		238	473	37	20
Cumul. costs	0–12	1818	809	0	12		2131	1323	0	21

Descriptive statistics of the effectiveness and cost outcomes.

^a^Observed means.

^b^Standard deviations.

^c^Number of observations with zero costs.

^d^Number of observations with missing values.

The DSQ was associated with several baseline variables, which were also predictors of the effectiveness measure or the costs. The potential confounders in modelling both effectiveness and costs were gender, ‘psychiatric diagnostic category on Axis I’ (DSM IV) [18], ‘IIP-C, total score’ [19], ‘SOC, Sense of Coherence scale’ [20] and ‘SAS-SR, work subscale’ [21] (Table 2). These potential confounders were chosen based both on a priori judgement on psychology and on Kendall’s *τ* with DSQ (p-value smaller than 0.1), and with the AUC or with DCP (p-value smaller than 0.2) (data not shown). Also, we have applied baseline adjustment of the effectiveness by adding the baseline GSI value in the effectiveness model [22]. Due to randomization, there was no association between therapy group (the intervention) and DSQ, thus the therapy group was not included in the model.

**Table 2:** Descriptive statistics of confounders

	**DSQ, immature defence styles**			
	**< 4 (N = 93)**		**≥ 4 (N = 100)**	
**Confounder**	**Mean**	**SD**	**Mean**	**SD**
Baseline GSI	1.05	0.44	1.49	0.49
Gender				
● Man	0.18	0.39	0.33	0.47
● Woman	0.82	0.39	0.67	0.47
DSM-IV, psychiatric diagnostic category on Axis I				
● Mood disorder only	0.44	0.5	0.58	0.50
● Anxiety disorder only	0.23	0.42	0.14	0.35
● Comorbid mood and anxiety disorder	0.33	0.47	0.28	0.45
● Other disorder	0.0	0.0	0.0	0.0
● No diagnosis	0.0	0.0	0.0	0.0
IIP-C, total score	7.46	2.92	10.17	2.63
SOC, Sense of Coherence scale	12.33	1.91	10.25	1.63
SAS-SR, work subscale	1.97	0.49	2.33	0.56

Observed means or prevalences, and standard deviations (SD) of the confounders.

### Models

The basic bootstrap method, which did not adjust for confounders, was compared with two model-based approaches, which adjust for confounders by using regression models. The first model-based method was based on cumulative measures of cost and effectiveness as outcomes, multiple imputation, bootstrap, generalized linear models and frequentist inference. The other method was based on hierarchical regression modelling of GSI and DCP at each MP using the Bayesian inference. These latter methods are described below, and referred to as the frequentist model and the Bayesian model although the models were not restricted to any particular inferential paradigm.

#### *Multiple imputation and bootstrap methods*

Multiple imputation [9] of the missing data was performed using procedure MI of the Sas System 9.2 [23]. The numerical Markov chain Monte Carlo (MCMC) method of this procedure was chosen because the missing data pattern was not monotonic. The MCMC method, in which the variables of the imputation model were assumed to be multinormally distributed, was applied separately for the DSQ groups. Effectiveness GSI was imputed for the repeated measurement data, and after the imputation the AUC was constructed from the imputed values using equation (4) given in the Appendix. The DCP were log-transformed in the MI. Auxiliary variables were not included in the imputation model due to convergence problems of the EM algorithm, which was used to estimate the initial values of imputation model parameters, of procedure MI.

In order to calculate the confidence intervals, the bootstrap method [1] was applied with 500 bootstrap samples. Multiple imputation was performed as a single imputation separately for each bootstrap sample [24].

#### *Models for cumulative outcomes with frequentist inference*

The observed values of the cumulative costs variable were all positive in this case, and a gamma regression model was therefore applied as the analysis model for the cumulative costs. The AUC was modelled using a linear regression model.

#### *Bayesian model for repeated measurement data*

The Bayesian method [8] for costs was based on a two-part hierarchical model because there were a considerable number of zero costs in the MP-specific cost data. The costs of patient *i* at MP *k* were factored as a product of two terms: $C_{\text{ik}}=C_{\text{ik}}^{Z}C_{\text{ik}}^{P}$. The first term in the product had a value of one if the costs were positive between MP’s *k*-1 and *k*, and zero if no costs had been incurred. A logistic regression model for the binary outcome $C_{\text{ik}}^{Z}$ was defined as

(1) $$P\;\left\{C_{\text{ik}}^{Z}=1\left|\;\beta_{k}^{Z},X_{\text{ik}}\right)\right\}=\frac{1}{1+\;\text{exp}\;\{-X_{\text{ik}}\beta_{k}^{Z}\}},$$

where *X*_*ik*_ denoted the row vector of the intercept, the confounders, MP *k* and the binary DSQ group indicator DSQ_*i*_. $\beta_{k}^{Z}$ were the corresponding regression coefficients, and $\exp\{\overset{Z}{\underset{k}{\beta }}\}$ were the corresponding odds ratios (OR).

The second term of the positive costs $C_{\text{ik}}^{P}$ was defined in a similar fashion. Because the distribution of the positive costs was skewed, the Gamma distribution was applied.

(2) $$C_{\text{ik}}^{P}∼\text{Gamma}\left(\exp\{X_{\text{ik}}\beta_{k}^{P}+U_{i}^{P}\}\tau_{{\text{DSQ}}_{i}k},\;\tau_{{\text{DSQ}}_{i}k}\right).$$

Random effect $U_{i}^{P}$ was individual. The regression coefficients represented the proportional changes in the expected value of the positive outcomes, i.e. a one-unit increase in the value of a covariate corresponded to a exp{*β*}-fold increase in the expected value. The possible heteroscedasticity was accounted for by allowing dispersion (inverse of variance-to-mean ratio) parameter *τ*_DSQ_*ik* vary over the DSQ groups and the MPs. Note that if *τ*_DSQ_*ik* = 1 then the expected value of the positive costs was equal to the variance, and if *τ*_DSQ_*ik*>1 then the variance was smaller.

Effectiveness *E*_*ik *_was modelled by using a linear, hierarchical model:

(3) $$E_{\text{ik}}=X_{\text{ik}}\beta_{k}^{E}+U_{i1}^{E}+U_{i2}^{E}t+{∊}_{\text{ik}}^{E},$$

where *t* is the follow-up time at MP *k*. There was assumed to be an individual linear trend, which was modelled by the random effects part $U_{i1}^{E}+U_{i2}^{E}t$.

The prior distributions of the regression coefficients $\beta_{k}^{\cdot }$ and the residual error terms ${∊}_{\text{ik}}^{E}$ were N(0, 100) and N(0, *σE*,DSQ_*i*_*k*2), respectively. The variance parameters $\sigma_{E,\cdot ,\cdot }^{2}$ and the dispersion parameters *τ*_·,·_ were distributed as InverseGamma(3,1) and Gamma(3,1)*a priori*, respectively.

The possible associations between measurement points, and the costs and the effects were accounted for using hierarchical models. The prior distribution for the three random effects were $U_{i}:={\left(U_{i1},\;U_{i2},\;U_{i3}\right)}^{T}:={\left(U_{i1}^{E},\;U_{i2}^{E},\;U_{i}^{P}\right)}^{T}∼N(0,\;\Sigma_{U})$ and for the corresponding covariance matrix *Σ*_U_=(*σ*_*ij*_)_*i*,*j*_∼InvW_10_(*I*_3_), where InvW_10_ was an abbreviation for the inverse Wishart distribution with 10 degrees of freedom and *I*_3_ the 3×3 identity matrix. Thus the correlation between the random effects was assumed to be zero *a priori*, and the costs and effectiveness were therefore assumed to be independent given the observed background information and the random effects. The correlation coefficients of the distribution of the random effects were defined as $\rho_{\text{ij}}:=\sigma_{\text{ij}}/\sqrt{\sigma_{\text{ii}}\sigma_{\text{jj}}}$.

The details of the model specifications, estimation, data augmentation and the model assessment are described below.

### Mathematical description of the model

#### *Notations*

Let i∈{1,2,…,n}=:I index the *n*=198 subjects and *k*=1,2,…,*K*:=5 the *K* intervention points where the measurements were made. Let time *t*(1)≡0 denote the baseline and *t*(*K*)=12 the duration of the follow-up in months. As there was some individual variation in the times when the actual measurements were made, let $t_{i}^{*}(k)$ denote the actual *k*^th^ measurement time when the outcomes *C*_*ik *_(DCP during $\left(t_{i}^{*}(k-1),t_{i}^{*}(k)\right]$) and *E*_*ik *_(GSI) of subject *i* were recorded.

The cumulative cost variable was defined as the sum $C_{i}^{\text{Cum}}:=\sum_{k=2}^{K}C_{\text{ik}}$. The effectiveness AUC was defined as:

(4) $$E_{i}^{\text{AUC}}:=\frac{1}{t(K)}\overset{K}{\underset{k=2}{\sum }}\frac{E_{i,k-1}+E_{\text{ik}}}{2}\left(t(k)-t(k-1)\right).$$

The incremental cost-effectiveness ratio was defined as

(5) $$\text{ICER}:=\frac{{\bar{C}}_{\text{DSQ}<4}^{\text{Cum}}-{\bar{C}}_{\text{DSQ}\geq 4}^{\text{Cum}}}{{Ē}_{\text{DSQ}<4}^{\text{AUC}}-{Ē}_{\text{DSQ}\geq 4}^{\text{AUC}}},$$

where ${\bar{C}}_{\cdot }^{\text{Cum}}$ and ${Ē}_{\cdot }^{\text{AUC}}$ are the DSQ group specific cumulative cost and effectiveness AUC means, respectively.

#### *Cumulative outcomes*

The model adjustment for controlling confounding was based on the ideas of Lee [6]. The individual predictions, which were based on the parameter values, the covariate values and the expected value based on the gamma regression model, were:

(6) $$E\;\left[C_{i}^{\text{Cum}}\left|\;\beta^{C},X_{i}^{C}\right)\right]:=\;\text{exp}\;\{X_{i}^{C}\beta^{C}\}\;\forall i,$$

where $X_{i}^{C}$ denotes the row vector of the covariates and *β*^C^ the corresponding column vector of the regression coefficients.

The analysis model for the effectiveness contains the group DSQ_*i *_and the confounders. The linear regression model was applied, and the individual predictions were:

(7) $$E\;\left[E_{i}^{\text{AUC}}\left|\;\beta^{E},\sigma^{2},X_{i}^{E}\right)\right]:=X_{i}^{E}\beta^{E}\;\forall \mathrm{i.}$$

The predicted margin [7] was the average of the predictions (6):

(8) $$PM^{C}(x^{\text{DSQ}}):=\frac{1}{n}\overset{n}{\underset{i=1}{\sum }}\;E\;\left[C_{i}^{\text{Cum}}\left|\beta^{C},\sigma^{2},X_{i}^{C,*}\right)\right].$$

In (8) $X_{i}^{C,*}$ was a modified version of the original covariate values. In this modification group variable DSQ_*i*_ was set to value *x*^DSQ^∈{DSQ <4, DSQ ≥4} for all patients *i* and the values of the other covariates remained at their original values. The adjusted difference of groups was difference PM^C^(DSQ <4)-PM^C^(DSQ ≥4). The predictive margins PM^E^(*x*^DSQ^) and the difference between the groups PM^E^(DSQ <4)-PM^E^(DSQ ≥4) were defined as in (8), but by using the individual predictions defined in (7). The adjusted ICER was calculated by using the predictive margins PM^E^(·) and PM^C^(·):

(9) $${\text{ICER}}^{\text{PM}}:=\frac{{\text{PM}}^{C}(\text{DSQ}\;<4)-{\text{PM}}^{C}(\text{DSQ}\;\geq 4)}{{\text{PM}}^{E}(\text{DSQ}\;<4)-{\text{PM}}^{E}(\text{DSQ}\;\geq 4)}.$$

#### *Repeated measurements*

In the Bayesian model, the posterior distribution was proportional to the product of the likelihood terms based on equations (1), (2) and (3), the joint density of the random effects and the joint density of all model parameters (denoted here by *θ*):

(10) $$\begin{array}{l} \;p\left(\left(\theta \;\right|\text{data}\right)\propto \\ \left[\underset{i,k}{\prod }P\left\{\left(C_{\text{ik}}^{Z}\;\right|\;\beta_{k}^{Z},\;X_{\text{ik}}^{Z}\right\}p\left(\left(C_{\text{ik}}^{P}\;\right|\;X_{\text{ik}}^{P},\;\beta_{k}^{P},\;U_{i}^{P},\;\tau_{{\text{DSQ}}_{i}k}\right)\right]\\ \;\times \left[\underset{i,k}{\prod }p\left(\left(E_{\text{ik}}\;\right|\;X_{\text{ik}}^{E},\;\beta_{k}^{E},\;U_{i}^{E},\;\sigma_{E,{\text{DSQ}}_{i}k}^{2}\right)\right]\\ \;\times \left[\underset{i}{\prod }p\left(\left(U_{i}\;\right|\;\theta \right)\right]\times p\{\theta \}. \end{array}$$

The predictive distribution of the outcomes for a set I of hypothetical subjects *i*^∗^ was defined as

(11) $$\begin{array}{l} \;\left[{\left(C_{i^{*}k}^{Z},\;C_{i^{*}k}^{P},\;E_{i^{*}k}\right)}_{i^{*}\in I}\;|\;\text{data}\right]\\ =\int p\left(\left(\theta \;\right|\;\text{data}\right)\underset{i^{*}}{\prod }p\left(\left(U_{i^{*}}\;\right|\;\theta \right)\\ \;\times \underset{k}{\prod }P\left\{\left(C_{i^{*}k}^{Z}\;\right|\;\beta_{k}^{Z},\;X_{i^{*}k}^{Z}\right\}p\left(\left(C_{i^{*}k}^{P}\;\right|\;X_{i^{*}k}^{P},\;\beta_{k}^{P},\;U_{i^{*}}^{P},\;\tau_{{\text{DSQ}}_{i^{*}}k}\right)\\ \;\times p\left(\left(E_{i^{*}k}\;\right|\;X_{i^{*}k}^{E},\;\beta_{k}^{E},\;U_{i^{*}}^{E},\;\sigma_{E,{\text{DSQ}}_{i^{*}}k}^{2}\right)\;dU_{i^{*}}\;d\mathrm{\theta .} \end{array}$$

Predictive distributions (11) were applied to calculate posterior predictive expectations and quantile points of functionals of

$${\left(C_{i^{*}k}^{Z},\;C_{i^{*}k}^{P},\;E_{i^{*}k}\right)}_{i^{*}\in I,\;k},$$

such as PM^C^(*x*^DSQ^) in (8) or the ICER ^PM^ in (9).

### Estimation

The frequentist regression analyses were performed using the SAS System 9.2 [23] procedures Genmod and Mixed for cumulative costs and AUC, respectively.

The Bayesian analyses were conducted using the OpenBugs software [13], which applies MCMC methods. The data management and the predictive distributions were handled using the R software [25].

#### *Data augmentation to handle missing data*

Predictive distributions (11) were also applied in the data augmentation procedures to handle missing outcome values. The corresponding predictive distribution for the missing baseline covariate value *X*_*ij*_, which belongs to at least one of the covariate vectors $X_{\text{ik}}^{Z}$, $X_{\text{ik}}^{P}$ or $X_{\text{ik}}^{E}$, was

(12) $$\begin{array}{l} \left[X_{\text{ij}}|\text{data}\right]\propto \\ \int p\left(\left(\theta \;\right|\;\text{data}\right)\\ \times \;p\;\left(\left(X_{\text{ij}}\;\right|\;\theta \right)\int p\left(\left(U_{i}\;\right|\;\theta \right)\underset{k}{\prod }P\left\{\left(\overset{Z}{\underset{\text{ik}}{C}}\;\right|\overset{Z}{\underset{k}{\beta }},\;\overset{Z}{\underset{\text{ik}}{X}}\right\}\\ \times \;p\;\left(\left(C_{\text{ik}}^{P}\;\right|\;X_{\text{ik}}^{P},\;\beta_{k}^{P},\;U_{i}^{P},\;\tau_{{\text{DSQ}}_{i}k}\right)\\ \times \;p\;\left(\left(E_{\text{ik}}\;\right|\;X_{\text{ik}}^{E},\;\beta_{k}^{E},\;U_{i}^{E},\sigma_{E,{\text{DSQ}}_{i}k}^{2}\right)dU_{i}d\mathrm{\theta .} \end{array}$$

The prior distribution for the discretized baseline DSQ was defined as Bernoulli(*p*^DSQ^), where the hyperprior for *p*^DSQ^ was chosen to be Uniform(0, 1). The other baseline covariates *X*_*ij *_having missing values were continuous, and they were assigned N(*μ*_*j*_, 1/*τ*_*j*_) priors with hyperpriors *μ*_*j*_∼N(0, 1000) and *τ*_*j*_∼Gamma(2, 1). As the number of missing values in these covariates was small, we chose not to elaborate the prior distributions further.

#### *Convergence checks of MCMC and assessment of model assumptions*

Two parallel chains were simulated with 60,000 iterations in addition to 10,000 iterations of burn-in in both chains. The chains were thinned by factor 15 resulting in 4,000 sampled values for both chains. Autocorrelations vanished quickly, which suggests good convergence (data not shown).

The Bayesian model was assessed using the Q-Q plots of standardised predictive errors (SPE). A total of 40 subjects denoted by set $I^{*}$ were excluded from the data, and the predictions for these 40 subjects were calculated based on their baseline information and all observed information of the remaining subjects in $I\cap {I^{*}}^{C}$. The SPE was defined as

(13) $$\begin{array}{l} \;E\left[\frac{E_{i^{*}k}-E_{i^{*}k}^{\text{Obs}}}{\sigma_{\text{E},∊}}|\text{data}\;\left\{I\cap {I^{*}}^{\text{C}}\right\}\right]\\ =\int \cdots \int \frac{E_{i^{*}k}-\overset{\text{Obs}}{\underset{i^{*}k}{E}}}{\sigma_{\text{E},∊}}p\left(E_{i^{*}k}|\overset{\text{E}}{\underset{i^{*}k}{X}},\;\overset{\text{E}}{\underset{k}{\beta }},\;\overset{\text{E}}{\underset{i^{*}}{U}},\;\overset{2}{\underset{\text{E},∊,{\text{DSQ}}_{i^{*}}}{\sigma }}\right)\\ \;\times p\;\left(U_{i^{*}}\;|\;\theta \right)p\;\left(\left(\theta \right|\text{data}\;\{I\cap {I^{*}}^{\text{C}}\}\right)\;dE_{i^{*}k}\;dU_{i^{*}}\;d\theta \;\;\forall i^{*}\in I^{*} \end{array}$$

where $E_{i^{*}k}^{\text{Obs}}$ denotes the observed value of effectiveness GSI. A similar approach for the positive costs $C_{\text{ik}}^{P}$ was applied.

## Results

The adjusted absolute differences of the cumulative costs (-161 and -166 based on the frequentist and Bayesian methods, respectively) appeared to be smaller than the unadjusted difference of -309 based on the bootstrap method (Table 3). The unadjusted difference was weakly significantly different from zero (P-value 0.094) whereas the corresponding adjusted difference based on the frequentist model was not significant (P-value 0.432). The Bayesian method, which modelled the measurement points separately, appeared to produce slightly lower predictive cumulative cost means than the frequentist predictive margins method. The estimates of the difference were close to each others.

**Table 3:** The observed and model-based estimates

	**Cumulative Cost**			**........... Effectiveness (AUC)...........**			
	**DSQ <4**	**DSQ ≥4**	**Δ**	**DSQ <4**	**DSQ ≥4**	**Δ**	**ICER**
Observed^**a**^ mean	1818	2131	-313	0.68	1.06	-0.38	830
Observed SD	809	1323	NA^**e**^	0.41	0.52	NA^**e**^	NA^**e**^
Observed CI	(1639, 1997)	(1835, 2428)	(-657, 31)	(0.59, 0.78)	(0.94, 1.18)	(-0.53, -0.22)	NA^**e**^
Observed p-value^**f**^	NA^**e**^	NA^**e**^	0.074	NA^**e**^	NA^**e**^	0.00	NA^**e**^
Bootstrapped^**b**^ mean	1838	2147	-309	0.72	1.09	-0.37	816
Bootstrapped SE	99	160	190	0.05	0.06	0.08	498
Bootstrapped CI	(1670, 2046)	(1855, 2469)	(-665, 47)	(0.61, 0.81)	(0.98, 1.2)	(-0.52, -0.23)	(-153, 1820)
Bootstrapped p-value^**f**^	NA^**e**^	NA^**e**^	0.094	NA^**e**^	NA^**e**^	0.00	0.098
Adjusted^**c**^ mean	1912	2073	-161	0.89	0.94	-0.04	-3569
Adjusted SE	126	152	208	0.05	0.06	0.08	102341
Adjusted CI	(1678, 2181)	(1799, 2362)	(-582, 224)	(0.79, 0.99)	(0.82, 1.04)	(-0.19, 0.1)	(-30215, 35896)
Adjusted p-value^**f**^	NA^**e**^	NA^**e**^	0.432	NA^**e**^	NA^**e**^	0.566	0.772
Predictive^**d**^ mean	1862	2028	-166	0.9	0.93	-0.03	-11777
Predictive SD	111	126	170	0.09	0.09	0.12	933871
Predictive CrI	(1656, 2090)	(1795, 2292)	(-501, 171)	(0.71, 1.08)	(0.75, 1.11)	(-0.26, 0.2)	(-26435, 23285)
Predictive p-value^**g**^	NA^**e**^	NA^**e**^	NA^**e**^	NA^**e**^	NA^**e**^	NA^**e**^	NA^**e**^

The observed and model-based estimates of cumulative costs and effectiveness measured by the area under the curve (AUC).

^**a**^The observed group means, and their standard deviations (SD) and group differences are not adjusted for confounders. 95% confidence intervals (CI) and tests were based on the t-test.

^**b**^The bootstrapped group means and differences, and their standard errors (SE) are based on multiply imputed and bootstrapped data but unadjusted for confounding.

^**c**^The adjusted group means and their differences are based on regression modelling. The rows marked adjusted mean and SE are based on predictive margins, frequentist inference, bootstrap and multiple imputation.

^**d**^The rows denoted by predictive means and SD are based on the hierarchical Bayesian model, posterior predictive group means and their differences, and the corresponding SDs and 95% credible intervals (CrI).

^**e**^NA corresponds to not applicable results.

^**f**^P-values correspond to the null hypothesis “no difference between groups” or “ICER equals zero”.

^**g**^P-values are generally not sensible in Bayesian inference, thus they are not reported.

The AUC differences for effectiveness were highly significant for the unadjusted bootstrapped difference (-0.38), but the adjusted mean -0.04 based on predictive margins was not significant and close to zero (P-values 0.000 and 0.566, respectively), whereas the predictive mean difference based on the Bayesian inference produced a much smaller difference of -0.03 with credible interval (CrI) (-0.27, 0.20) (Table 3).

The unadjusted ICER estimate was slightly higher than the adjusted estimate (Table 3). The standard errors, however, were large. The unadjusted ICER was weakly significant (P-value 0.098), but the adjusted ICER was not significant (P-value 0.772). The adjusted ICER and the posterior expectation of the ICER were not applicable, because the denominator of the ICER (the effectiveness difference) was near zero, in which case the ICER was undefined [2].

**Figure 1:**
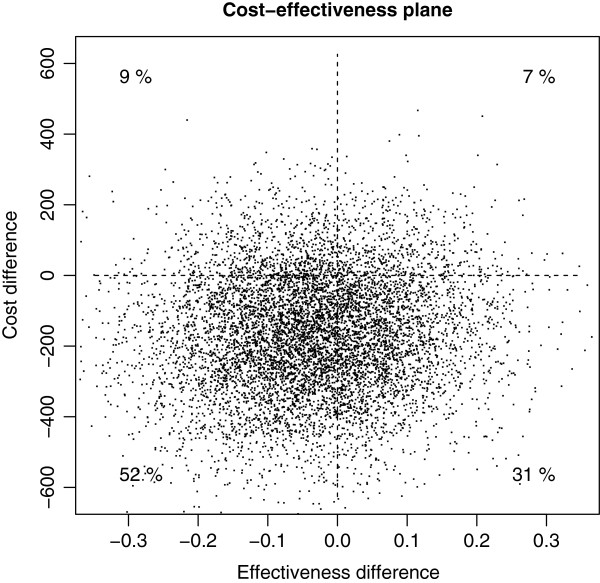
**The cost-effectiveness plane.** The Bayesian method was applied to calculate the posterior predictive differences of effectiveness means ${Ē}_{\text{DSQ}\;<4}^{\text{AUC}}-{Ē}_{\text{DSQ}\;\geq 4}^{\text{AUC}}$ and cost means ${\bar{C}}_{\text{DSQ}\;<4}^{\text{Cum}}-{\bar{C}}_{\text{DSQ}\;\geq 4}^{\text{Cum}}$.

The cost–effectiveness plane shows that in the group where DSQ ≥4 both the costs and the symptoms were on a higher level than in the group where DSQ <4 (Figure 1).

Due to the large number of parameters in the regression models defined above, which were merely a technical utility as the objective was to adjust the average costs and effectiveness for possible confounding, we focus on a limited number of parameters of the Bayesian model in the following.

There were significant correlations between average levels of individual costs and effectiveness over time (posterior expectation $E[\rho_{1,3}|\text{data}]=0.32$ and CrI (0.00,0.57)), and the individual slope of effectiveness ($E\;[\rho_{1,2}|\text{data}]=-0.17$ CrI (-0.33, -0.00)), which cannot be explained by the background information included in the regression models. The correlations between the intercept of positive costs, and the slope of effectiveness, however, were practically zero ($E\;[\rho_{1,3}|\text{data}]=-0.03$).

The dispersions of the positive costs showed considerable variation over the measurement points, but the residual variances of effectiveness were close to each other (Table 4). During the first measurement interval the positive costs varied relatively little ($E\;[\tau_{\cdot ,2}\;|\text{data}]$ were large) because the majority of patients took the study treatments, each of which lasted approximately six months and was covered by the first two measurement intervals. Some patients took auxiliary therapies or other psychiatric treatments both during and after the study therapies, which resulted in higher costs, whereas other patients took no auxiliary treatments, which resulted in lower costs. This resulted in greater individual variation during the last measurement interval ($E\;[\tau_{\cdot ,5}\;|\text{data}]$ were small).

**Table 4:** Variance parameter estimates

	**DSQ < 4**			**DSQ ≥ 4**		
***θ***^**a**^	$E{[\theta |d]}^{b}$ ^**b**^	**95% CI**^**b**^		$E{[\theta |d]}^{b}$ ^**b**^	**95% CI**^**b**^	
$\sigma_{E,\cdot ,1}^{2}$	0.10	0.07	0.14	0.13	0.09	0.19
$\sigma_{E,\cdot ,2}^{2}$	0.09	0.06	0.12	0.12	0.09	0.18
$\sigma_{E,\cdot ,3}^{2}$	0.07	0.05	0.11	0.09	0.06	0.13
$\sigma_{E,\cdot ,4}^{2}$	0.10	0.06	0.16	0.17	0.1	0.26
*τ*_P,·,1_	12.15	8.28	17.19	8.09	5.55	11.37
*τ*_P,·,2_	6.07	4.06	8.51	3.79	2.61	5.3
*τ*_P,·,3_	4.12	2.57	6.08	4.67	3.01	6.82
*τ*_P,·,4_	2.07	1.21	3.13	1.95	1.16	2.91

The parameters controlling the variance of effectiveness and the dispersion of the positive costs in the Bayesian model.

^a^Parameters $\sigma_{E,\cdot ,\cdot }^{2}$ control the variance of the effectiveness measure, and parameters *τ*_·,·_ (inverse of variance-to-mean ratio) the dispersion of the positive costs in the Bayesian model. Parameter *θ* is a generic notation representing one of the parameters $\sigma_{E,\cdot ,\cdot }^{2}$ or *τ*_·,·_.

^b^The point estimates are posterior expectations E[θ|d] and also 95% credible intervals (CI) are presented

**Figure 2:**
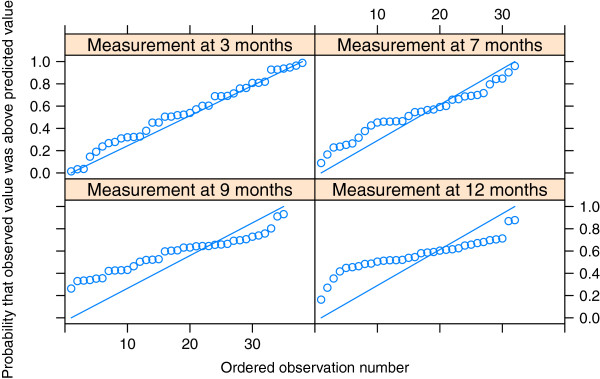
**Q-Q plots of effectiveness outcome GSI.** The four measurement points at 3, 7, 9 and 12 months were used.

**Figure 3:**
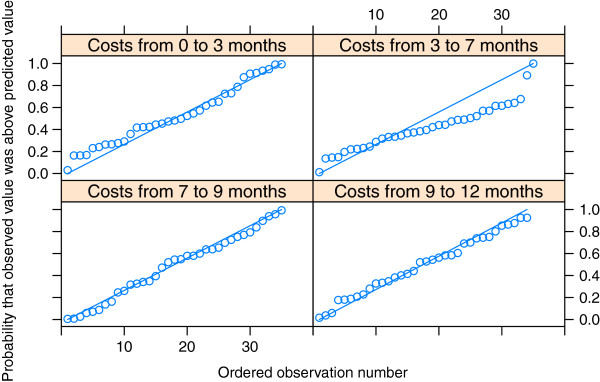
**Q-Q plots of positive costs.** The measurement intervals were 0–3, 3–7, 7–9 and 9–12 months. The measurements with zero costs were excluded.

The posterior predictive checks based on the Q-Q plots showed that the model for costs fitted well for intervals 0 to 3 months and 3 to 7 months, but after that the predictions were too low. The model for effectiveness did not fit well at the last two measurement points (Figures 2 and 3).

## Discussion

This paper compared the standard bootstrap-based method in a cost-effectiveness analysis with two model-based methods based on the frequentist and the Bayesian inferences. The skewed, non-negative distribution of cost variables was analysed using the gamma regression and the two-part models, and model adjustment for potential confounding was performed using the predictive margins and predictive distributions in the frequentist and the Bayesian inferences, respectively.

The benefit of using the predictive margins method or the predictive distributions of the Bayesian inference to calculate the adjusted averages of costs and effectiveness is that there is that also other link functions than the identity function can be used in regression modeling. For example, Nixon and Thompson [5] applied identity link functions both for gamma distribution of the costs and for the normal distribution of the effectiveness. The average of the covariates over all individuals multiplied by the corresponding regression coefficients were restricted to zero in order to interpret the intercept terms as the group averages. There is no need to make such restrictions or to stick with the identity link function when using predictive margins or Bayesian predictive distributions. Other link functions, which are commonly used in generalized linear models, can be applied with these methods as well thus avoiding the possibility of negative linear predictors (and predictive values) in case of the gamma distribution.

Both methods based on the regression models could have been applied in the case of repeated measurements by using either frequentist or Bayesian inference. However, the prevalence of zero costs during the first two follow-up periods was very low, thus an application of a logistic regression model and frequentist inference in the two-part model was not plausible. Furthermore, application of a joint hierarchical model would have been difficult because effectiveness and positive costs were modelled using different families of distributions, normal and gamma distributions, respectively. The Bayesian method avoided the numerical instabilities by using informative prior distributions for the regression coefficients and the missing data values were augmented during the MCMC simulation in a straightforward manner.

Bayesian model averaging [26,27] has been proposed to handle the skewness of cost variables or for the selection of important predictors, respectively, but simultaneous handling of skewness, heteroscedasticity and adjustment for confounding factors can be challenging. We modelled the heteroscedastic residual variance of the cost variables in the case of the repeated measurement data, which allows the model to adapt to the distribution of the costs. The posterior predictive checks suggested that the model fit was good.

The predictive margins approach for the cumulative outcomes was flexible, and the procedure described in this paper can be extended to the case of zero costs and two-part models.

Olsen and Schafer [28] introduced a model with random effects for both parts of the two-part model for costs. They reported that the random effects were usually correlated. In our study, however, the sample size was considerably smaller and the proportion of zero costs was very low during the first seven months of follow-up. Therefore, application of separate random effects for the zero costs (and possible positive correlation with the random effect(s) of the positive costs) was suppressed.

Potential extensions of the model include, for example, using a higher order random effects model for positive costs and adding a random effect to the logistic model of zero costs. These extensions would, however, require more measurements or more individuals. Another extension of the model would be to handle the skewed distribution of the effectiveness outcome, especially at the later stages of the follow-up by using, for example, the skewed normal distribution [29].

The patient groups were not defined using (randomized) intervention groups, which is the case in most cost-effectiveness analyses, but with the DSQ, which complicates the interpretation of the ICER statistic. The DSQ is a rather stable characteristic of a patient, which cannot be altered by a researcher, whereas interventions in general can be altered. The interpretation of the ICER statistic is the change in average costs per one unit in the change of the average effectiveness measured in terms of the AUC. It is also important to bear in mind that in this work large values of the effectiveness outcome GSI correspond to more severe symptoms (less benefits) whereas in commonly used effectiveness outcomes such as the quality adjusted life years (QALY) large values correspond to greater benefits. Therefore the usual interpretation of the quadrants of the cost-effectiveness plane[30] is reversed with respect to the vertical axis. In this case the unadjusted ICER estimates were positive indicating that in the group DSQ¡4 the effectiveness was better and the costs were lower.

Our results allow a decision-maker to assess the importance of patient characteristics such as the DSQ in this study. If a patient in the group DSQ ≥4 become similar to a patient in the group DSQ¡4, the cost would decrease by 816 euros (the bootstrapped point estimate) per one unit decrease in the AUC based on the GSI on average. However, the adjusted estimates indicate that not only the effectiveness difference but also the cost difference vanished thus it is not reasonable to present such a standardized estimate of the change in costs as the ICER.

Limitations of the proposed methods are mainly related to the various modelling assumptions, but there are few alternatives to parametric models in order to adjust for confounders. For example, the effectiveness measure was nonnegative, but the model was based on normality assumptions, thus predictive effectiveness values can be negative. The poor performance of the effectiveness model was likely to be due to the skewed distribution, which was not accounted for using a model based on normal distribution. SCL-90-GSI can have only positive values, and the reduction in symptoms caused a considerable proportion of observations to lie close to zero. Linearity assumptions or possible interactions were not tested, but could be done in future work.

## Conclusion

Our paper demonstrates how to combine several methods for performing cost-effectiveness analyses in observational studies, which are often subject to effects of confounding and missing data. Our results based on regression modelling confirmed that there was a need to adjust for the confounders in this study, thus the standard unadjusted methods based on the bootstrap method, were not adequate. Unadjusted methods showed significant differences between the groups, but the adjustment for confounders showed no the significant differences thus yielding different conclusions. Not all associations between the costs and effectiveness could not, however, be explained by the observed confounders only, thus the hierarchical model showed clear non-zero correlations between the random effects. The OpenBUGS code is available from the corresponding author upon request.

## Competing interests

The authors declare that they have no competing interests.

## Authors’ contributions

PK, TH and TM conceived the study. TH developed the methods, conducted the analyses and wrote the manuscript. TM provided expertise in cost-effectiveness analyses. OL and PK provided expertice in psychotherapy. OL, PK ja EV provided the data. EV programmed the SAS macro. All authors read and approved the final manuscript.
